# Digital Health Rehabilitation Can Improve Access to Care in Spinal Cord Injury in the UK: A Proposed Solution

**DOI:** 10.5195/ijt.2020.6312

**Published:** 2020-06-30

**Authors:** Anbananden Soopramanien, Shiva Jamwal, Peter W. Thomas

**Affiliations:** 1 Centre of Postgraduate Medical Research and Education, Bournemouth University, Bournemouth, United Kingdom; 2 National Spinal Injuries Centre, Stoke Mandeville Hospital, Aylesbury, UK; 3 Department of Medical Sciences and Public Health, Bournemouth University, United Kingdom

**Keywords:** Digital Health, Multidisciplinary Team, Spinal Cord Injury, Rehabilitation, Telehealth, Telemedicine, Telerehabilitation

## Abstract

Lack of specialist beds, inadequate finance and shortage of skilled staff make it difficult for Spinal Cord Injury Centres (SCICs) in the United Kingdom (UK) to admit all newly injured individuals. Length of stay of those admitted can be too brief. At discharge, follow-up care is sparse and inadequate. We therefore propose that specialist spinal units redefine their roles and act as catalysts to build capacity by enhancing expertise in the wider community. SCICs can devolve certain tasks locally to less specialised units with their support, training, and guidance. This Commentary further proposes that use of Digital Health Technologies, (i.e., to deploy telemedicine, telehealth, and telerehabilitation), can enhance rehabilitation opportunities. The authors set-forth their vision for a comprehensive web portal that will serve as a primary resource for evidence-based practice, information on guidelines, care pathways, and protocols of SCI management. At any stage during the acute management of SCI and following discharge, rehabilitation specialists could conduct remote consultation with persons with SCI and acute care specialists via the web portal, allowing timely access to specialist input and better clinical outcomes. The proposed portal would also provide information, advice and support to persons with SCI and their family members. The strategic use of digital health technologies has been shown to result in cost and time savings and increase positive outcomes.

Digital health technologies have the potential to fill a significant void in the provision of rehabilitation as Spinal Centres across the United Kingdom (UK) struggle to find a solution to the lack of access to neurorehabilitation and are unable to deliver what Sir Ludwig Guttman had advocated. The latter, a neurosurgeon, opened the National Spinal Injuries Centre (NSIC) at Stoke Mandeville Hospital in Aylesbury on 01^st^ February 1944. He introduced the concept of the total, comprehensive and integrated care with early intervention and long-term follow-up by a team of professionals with complementary skills (i.e., physicians, surgeons, nurses, and therapists). Prevention of Spinal Cord Injury (SCI) and management of complications soon became part of the strategy, as did return to work and the introduction of sports and exercises in active rehabilitation. His vision of the holistic management of SCI ‘from injury to grave' by a team of specialists founded the very principle of SCI Medicine (Schultke, 2001). Sir Ludwig Gutman's model of rehabilitation has been adopted throughout the world and holds credit even in today's time to deal with the compromise to the spinal cord that can come from a traumatic injury, vascular insult or a disease process (i.e., infection or tumour). This model takes into account that SCI management is complex and requires input from a team of highly skilled professionals offering specialist expertise and a care pathway.

However, from the late 1990's it was becoming obvious that individuals suffering from SCI in the UK would soon face significant difficulties with accessing rehabilitation and long-term follow-up care, due to the mismatch between increasing demand and dwindling resources. How could the situation be improved?

Access to European Union (EU) funding in 2003 made it possible for us to initiate a research study and explore how we could address these concerns. The THRIVE project (**T**elere**H**abilitation for spinal cord injured patients th**R**ough **I**nteractive **V**ideo **E**ndorsement) followed people after discharge from four European spinal units (Salisbury and London, UK; Montecatone, Italy; Brussels, Belgium). It was one of the earliest randomised controlled trials carried out in the field of telemedicine and we presented our findings in a publication (Dallolio et al., 2008). People with spinal cord injury were assigned to two groups, one of which used telemedicine to monitor their condition after discharge. Our study, based on results from one site, provided some of the first quantitative evidence, that telemedicine, as compared to standard care, may offer benefits in functional improvement to those discharged from a spinal cord unit. The Salisbury team carried out a qualitative analysis with input from Bournemouth University and confirmed a high acceptance level for the use of technology (Soopramanien et al., 2006) via the regular monitoring that telemedicine offered. We later produced a video on telerehabilitation (Soopramanien, 2013), based on another study, PEACEanywhere (“Technology Strategy Board,” Oct 2009 - Dec 2012) for which we analysed a Care Portal^®^ (Thomas et al., 2013) (manufactured and marketed by Docobo Ltd) and carried out extensive face-to-face and online surveys (Soopramanien, 2012; Soopramanien et al., 2013) to understand the needs of those affected by SCI and their relatives. Recently, there have been a few examples of telemedicine being used in Delhi at the Indian Spinal Injuries Unit (Tyagi et al., 2019) and Nepal, initiated by Leeds University (“Telerehabilitation in Nepal Now Allows Specialists to Link with Patients, Saving Time and Money,” 2020).

The technology we used for our previous research studies is now outdated as digital platforms are evolving and providing ever expanding options and possibilities. Digital health rehabilitation makes use of digital technologies (e.g., smart phones and mhealth, wearables, sensors for vital sign monitoring, artificial intelligence, etc.) to facilitate interaction between those affected by SCI, their relatives or carers and specialist clinicians. Time has now come to offer rehabilitation using the digital health platforms at the different stages of an individual's journey, from the onset of paralysis to return into the community and lifelong care. How could we deliver on these aspects of care?

## METHODS

To answer the above question, we have used our clinical experience gathered in the practice of rehabilitation in the UK, France, India, Peshawar (Pakistan/Afghanistan border), Romania and the main findings of the different research activities highlighted. The surveys mentioned above in the Introduction were led by two service users who worked closely with a team of researchers. To present evidence-based data, we carried out a literature search that yielded the references previously cited in this Commentary. Other references were equally relevant (Buckingham et al., 2016; Guo & Albright, 2018; Hill & Phillips, 2006; Hillier & Inglis-Jassiem, 2010; Jaglal et al., 2009; Laver et al., 2012; National SCI Strategy Board-Initial Management of Adults with Spinal Cord Injuries, 2012; Thompson, 2013; Wellbeloved-Stone et al., 2016). Finally, we have relied on our wide exposure to digital health technology since 2003. Our results are summarised below.

## RESULTS

### DESCRIPTION OF PRESENT MANAGEMENT

#### ACUTE MANAGEMENT

After an accident, people with SCI are referred to trauma centres or Major Trauma Networks (MTN) (Bonner & Smith, 2013). Within four hours of their admission, they are referred to a consultant at a linked Spinal Cord Injury Centre (SCIC). An optimum management plan is agreed between the treating consultant delivering acute care and the on-call Spinal Rehabilitation consultant. A dataset is entered on the SCI registry / database within 12 hours. The SCIC is expected to provide acute outreach (telephone and in-person) under established protocols that include support to staff, the newly injured and family within 5 days of injury.

#### REHABILITATION

Following the emergency care, individuals with SCI are transferred to acute rehabilitation settings. The venue is ideally an adequately resourced SCIC wherein staff have specialist training and skills in the management of SCIs. However, patients may stay longer in the MTN, due to shortage of acute rehabilitation beds and delays in admission (Rose, 2014). There are 12 SCICs across the UK that offer specialised rehabilitation care, of which eight are in England and provide a total of 395 rehabilitation beds (“Annual Statement - NHS Specialised Spinal Cord Injury Services,” 2017-19).

**Figure 1.**
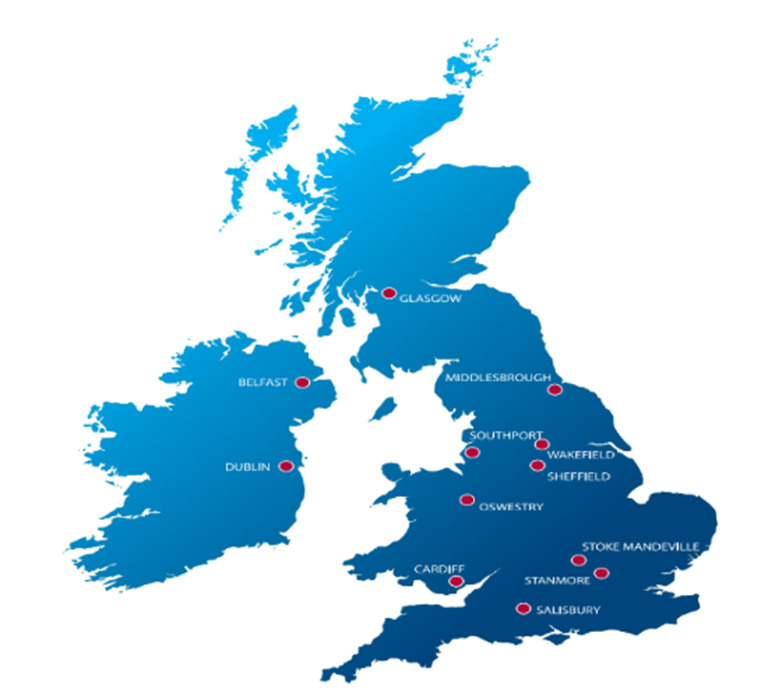
The location of Spinal Cord Injury Centres (SCICs) in UK (“Annual Statement - NHS specialised spinal cord injury services,” 2017-19).

Each centre, as shown in Figure 1 is linked to a MTN and has a defined catchment area. The centres have established relationships with clinical and social groups to provide effective service and facilitate community reintegration. Not everyone with a SCI is currently admitted to a specialist rehabilitation centre due to the lack of beds and many must continue their journey in non-specialist units or be discharged home.

During their rehabilitation, staff liaise with individuals with SCI to set SMART (Specific, Measurable, Achievable, Relevant and Time-bound) goals to deal with clinical issues, improve functional independence and prepare them for discharge to their home communities.

#### DISCHARGE PLANNING

For individuals with SCI, discharge planning is an important milestone in the journey to ensure a smooth transition from hospital to home and community.

#### LONG TERM MONITORING

After discharge many individuals with SCI will encounter numerous challenges with their bladder, bowels, skin, chest, and low mood (i.e., depression), such that they require long-term follow-up. Pressure injuries are frequent post-discharge.

Discharged individuals can be seen in the outpatient clinic for yearly monitoring. At that encounter they do not often see more than one professional (traditionally the consultant, but now increasingly a specialist nurse). Rarely would they meet the other members of the multi-disciplinary team at the same appointment. They may often be offered a further appointment to meet a physiotherapist or occupational therapist, implying yet another visit for an ‘in-person' contact. Alternatively *outreach* clinics after discharge are now being organised on a small scale in local communities. The purpose is to monitor complications linked not only to *ageing* related to normal physiology, but also due to the effects of SCI on tissues, organs and systems that have been deprived of normal innervation for weeks, months, or years.

In spite of all these limitations, the overall picture is encouraging. The regular follow-ups by spinal units, and improvement in SCI medicine have resulted in the quality of care being so much improved that the life expectancy of people with spinal cord injury has increased. Today, hardly anybody with SCI dies of renal failure.

For decades, SCIs in the UK maintained this legacy through their diligent work providing excellent rehabilitation and follow-up care. They were recognised as world leaders in the delivery of care. Those under their care could rely on the high standards of practice and support throughout their lives. Help was within easy reach, and individuals could be identified quite soon if they developed complications post-discharge.

Such an open-door policy was possible thanks to the vision of the spinal cord injury leaders, the relatively small number of individuals with SCI, bed capacities of the respective units and appropriate funding. Unfortunately, this ‘golden age' appears to have passed. Careful analysis of the challenges faced by spinal units is essential before discussing the overall picture and recommending potential solutions for the future, like Digital Health Rehabilitation.

### CURRENT CHALLENGES FACING SPINAL CORD INJURY

#### THERE IS PRESSURE ON BEDS

Only 395 beds are available to accommodate between 1200 and 2500 new spinal cord injury cases per year in England appropriate for admission to a SCIC (excluding cauda equina injury). According to the National Health Services (2019) Specialised Spinal Cord Injury Services Annual Report, (“Annual Statement - NHS Specialised Spinal Cord Injury Services,” 2017-19) there were 2,429 new referrals made to the eight Specialist SCI Centres in England in 2017-2018, while 11,767 outpatient attendances and 1,420 secondary admissions were recorded in all eight SCI centres in this period. Approximately 600 individuals with possible acute SCI or cauda equina syndrome were managed as outpatients or supported by outreach services.

The Spinal Injuries Association, a national charity, reported in 2008 that 10% of its 829 members had never been under the care of a SCIC. Patients with cauda equina have told us that they may be seen once in the spinal cord injury outpatient setting and never offered any follow-up appointment. Yet, individuals with SCI in the community are not equipped to deal with bowel and bladder incontinence.

#### ADMISSIONS ARE DELAYED

The National SCI Database for the year 2013-14 recorded a range of 0-279 days from the time of referral to admission to a SCIC. Long delays in accessing specialist rehabilitation are accounted for by the inability to discharge patients early and the ‘labour-intensive' expert care that is required to look after the needs of a person with high-level paraplegia, tetraplegia, or a person with SCI requiring ventilatory support. Delayed admission to a SCIC can cause increased risks of avoidable secondary complications such as pressure injuries, contractures and infections; these can negatively impact rehabilitation and lead to a longer length of stay. In 2013-14, 8% of delayed admissions to SCIC had a history of pressure injuries, which could have been avoided.

#### THE NATURE OF INJURIES IS CHANGING

There is an increase in non-trauma referrals. The annual report on SCI 2017-18 states that 33% of patients referred to SCIC sustained their injury due to trauma; 60% of traumatic injuries resulted of falls, and 23% resulted from road traffic accidents. Non-trauma accounted for 67%. Whereas in 2013-14, 43% of SCI were recorded as non-traumatic (“Annual Statement - NHS Specialised Spinal Cord Injury Services,” 2017-19; Rose, 2014). The statistics indicate that non-traumatic SCIs have more than doubled since 2008, when only 21% of new injuries were non-traumatic (“SCI Service Specification No. 170119s,” 2019).

#### THE AGE OF ONSET OF SCI HAS INCREASED

In 2009 the range of SCI onset was 30-40 years, with the range's upper limits increasing to 65-69 years in 2017-18 (NHS, 2019). A significant proportion (44%) of persons with SCI are now over the age of 60 years. Also, it is worth noting that improved acute care (through the emergence of acute trauma centres and enhanced intensive care strategies) has resulted in 20% increased survival rate after catastrophic injuries, (Dryden et al., 2004) with a rising number of cervical flexion injuries, cauda equina, central cord syndromes, and ventilator dependency.

#### THE LENGTH OF STAY IS SHORTER

Depending on their level of injury, those admitted now have an average length of stay of 8 to 12 weeks at SCICs from the day of mobilisation, as opposed to 6 to 9 months of inpatient rehabilitation in the past. This shortened length of stay allows a higher throughput of patients, but one can question the wisdom of discharging individuals to unsafe environments or half-way through their rehabilitation process, whilst they may not be totally out of ‘spinal shock'.

#### POST-DISCHARGE FOLLOW-UP

The NHS Clinical Advisory Group recommends lifelong follow-up by the SCICs to minimise secondary complications, optimise potential of individuals with SCI, and improve quality of life (“NHS Service Standards for Patients Requiring Spinal Cord Injury Care,” 2010; “NHS Clinical Advisory Groups,” 2011). We are aware of the pressure that NHS Trust managers are exerting on clinicians locally to put an end to the lifelong follow-up and transfer such care to the district general hospitals. Service standards that exist in England (“NHS Service Standards for Patients Requiring Spinal Cord Injury Care - v7,” 2013) set out the services that must be provided and SCIC services are funded on that understanding; this includes outpatient review (section 10). The provision of services is arranged through NHS England Specialist Commissioning and there is an expectation of what must be provided (“NHS Standard Contract for Spinal Cord Injuries D13/S/a,” 2013). The readmissions and further services are funded through commissioning tariffs but are provided outside the initial management commissioning tariff. The availability and delivery of these services varies across the SCICs.

#### FINANCE

As per the national statistical report, despite rapidly increasing demand and struggling service providers, the UK's spend on long term care has consistently been 16% of the healthcare budget (which is only 1.8 per cent of GDP) from 2013 to 2017 (Stevens et al., 2017). In the present political and financial environment when governments are struggling to give a fair share of gross domestic product (GDP) to health services as a whole, rehabilitation medicine and services get an even smaller pot of funding, despite robust evidence on the cost-effectiveness and benefits of rehabilitation across whole healthcare. NHS England has a projected shortfall of £30 billion for health in 2020, besides the 12% budget cut in social services in the last four years, (George & Martin, 2014). COVID 19 may make availability of funds even more challenging.

The economic cost model for SCI in the UK by London School of Economics suggests that the overall cost per SCI in the UK is £1.12 million, ranging from £0.47 million per person with ASIA grade D injuries to £1.87 million per tetraplegia ASIA grade ABC injury (McDaid et al., 2019). The cost of a hospital inpatient stay at a SCI Centre in the UK is £495-£554 per day. Although direct medical and rehabilitation costs are increasing, the indirect costs associated with loss of productivity across the lifespan can exceed the direct costs. Despite the support and provisions by the government, not many individuals with SCI get back to work and become taxpayers. Vocational rehabilitation guidelines released in 2017 by Multidisciplinary Association of Spinal Cord Injury Professionals (MASCIP, 2017) state that only one third of persons with SCI go back to work. A study by Stoke Mandeville hospital found that only 33 per cent of their study participants were employed (Tasiemski et al., 2000).

#### WORKFORCE

**Figure 2.**
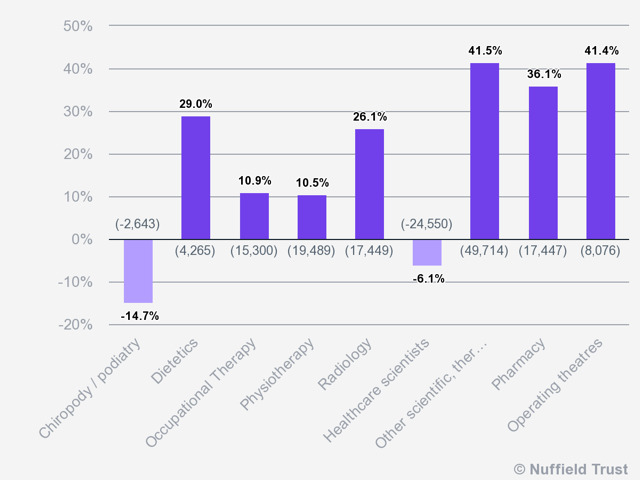
Percentage of change in fulltime equivalent, scientific, therapeutic and technical staff by specialty, 2009-2018. (Source: Nuffield Trust analysis of NHS Digital cited in Dayan & Palmer, 2018)

An acute shortage of clinical workforce is making the NHS increasingly unsustainable. Since 2009, there has been a considerable fall in nursing numbers in community health services. The numbers continued to decline by 37% between November 2009 to November 2018 (Dayan & Palmer, 2018; Rolewicz & Palmer, 2019). As per the OECD's report on long-term conditions (George & Martin, 2014), the UK has 2.8 doctors per 1,000 which are below the average of 3.6 for our peer countries, and substantially lower than in most developed countries. These increasing needs suggest that the UK requires an expansion of medical consultant and trainee numbers by at least 7% in the next two years, simply to stay at the current level of provision (Royal College of Physicians, 2010). Along with the shortage of medical consultants and nurses, allied health forces are also struggling to keep up the demand. There has been an average of only 18 per cent increase in therapeutic staff with an average of 10 per cent increase in Occupational Therapy and Physiotherapy numbers in the last decade. Figure 2, on the following page, depicts the change in the full-time equivalents for therapists between 2009-2018 (Dayan & Palmer, 2018).

## DISCUSSION

The data show that today, the SCI population is predominantly older, lives longer and has high-level and incomplete injuries. Patients have more complex medical needs as, at an older age, they may already have established comorbidities such as diabetes, cancer or cardiovascular disease. Furthermore, with an upward trend in life expectancy of the SCI population, we should prevent or manage the complications with the bladder, digestive system and bowels, skin, and conditions like autonomic dysreflexia and syringomyelia. With ageing, the SCI population is likely to become physically more dependent for transfers and activities of daily living. Thus, many professionals are required to deal with not only the existing medical and social requirements of individuals with SCI but also changing functional needs. Yet, there are constraints related to finance and human resources.

Numerous pressures are soaring as achieving good rehabilitation outcomes are costly and take time. The reality is that due to demands on rehabilitation beds, length of stay of the individuals with SCI is reduced considerably. The SCICs are under duress to discharge patients due to long waiting lists, and clinical commissioning groups (CCGs) are obliged to save costs, often resulting in a shorter period of rehabilitation. It is worth noting that many complications related to spinal cord injuries only surface 3-4 months post-injury (i.e., spasticity, neuropathic pain, etc.), when individuals may be just out of the ‘spinal shock' phase. They are discharged from the SCIC as their spasticity peaks or the bladder detrusor becomes hyperactive and they are left to manage on their own in the community. SCICs are not able to follow them up and general practitioners are not trained to deal with the complex needs linked to spinal cord injury.

The current situation is a downward spiral wherein decision-making bodies admit shortfalls but are not positioned to offer alternatives. The British Society of Rehabilitation Medicine (BSRM) recognises that most rehabilitation services are not adequately staffed or resourced to meet the proposed response times or standards. The situation will worsen if alternatives are not offered. As clinicians, our first instinct would have been to insist on the recruitment of more staff and building of more inpatient and outpatient facilities across the country by opening more spinal units and/or enhancing bed capacity in each existing unit. Sadly, the challenges are more complex than we have described them. It is therefore time to look at new ways of delivering healthcare and rehabilitation to continue to provide the quality of care to the spinal injured population.

Digital Health Rehabilitation (that includes telerehabilitation) can be an answer to the myriad of challenges faced by present health and rehabilitation services. It is not designed to replace in-person care but to complement the current system at each stage from prevention to acute and follow-up care, providing a smooth and timely access to the specialist input. The technology can also offer training and advice to the professionals, virtual and/or outreach clinics by the multidisciplinary team as well as research and long-term monitoring opportunities.

Limited budgets and finances as well as the clinical challenges that we have highlighted can be an opportunity for health professionals to think innovatively and review the way rehabilitation is delivered by the already stretched NHS. Long term rehabilitation services will need to be remodelled and better prepared for rapid changes. The NHS is encouraging clinicians to think differently, learn from other countries, and maximally exploit the already existing resources. Adopting technological advances in healthcare has been at the forefront of the NHS future plans. The King's Fund has indicated that in the wake of growing demands and pressure, health and social services will need to work differently, and technology will be one of the main enablers to deliver the much-needed change.

Technological advances, referred by the National Service Framework (NSF), will be of great benefit in the challenging financial environment. BSRM also strongly advocates the use of technology to provide greater opportunities for people with disabilities. It will have the potential to reach out to a significant number of people at the same time. It can be a solution to numerous issues faced by current health services. Digital health rehabilitation input can provide immediate and long-term solutions.

Topol's (2019) report on telemedicine acknowledged that digital medicine is increasingly empowering individuals to manage their health and wellbeing, transforming the traditional ‘patient-clinician' relationship. The report has reviewed NHS's initiative in telemedicine and digital health at different sites and services - fracture clinic, diabetes mellitus and COPD clinics and radiology clinic. Spinal Injury Rehabilitation would be a worthwhile addition.

## RECOMMENDATIONS

### DEVELOP A PROPOSED INFRASTRUCTURE TO SUPPORT DIGITAL TECHNOLOGIES IN REHABILITATION

The THRIVE study (Dallolio et al., 2008) demonstrated that telerehabilitation consultations could be an effective way to interact with staff, people with SCI and their relatives at all stages of their journey and follow-up in the community, provide ongoing support, monitor individuals' progress, and flag early signs of complications thereby reducing readmissions. Backed by the success of this study and that of the subsequent research project (“Technology Strategy Board,” Oct 2009 - Dec 2012), our team seeks to develop an app or web portal which will be supported by a pool of specialists in spinal cord injuries. The app will be downloaded from the App Store or Google Play. It is a web-based portal that will allow quick access to professionals via virtual clinics and remote consultations, at all stages of the service user's journey. This paper's first author has already started using his own app for e-neurorehab to follow patients in the community.

Thus, an app can be ‘tailor-made' for individual spinal units or offer a generic service ‘centrally,' (i.e., on behalf of all Spinal Injury Centres). Different and complementary systems could operate in parallel to attend to the needs of the wider population of people with spinal cord injury, living at home. The ‘app' will be a primary source of comprehensive training and information; tailor-made education packages and evidence-based practice on spinal cord injury management will be offered. It will have two domains: healthcare professionals, individuals with SCI and caregivers. Education/training will be provided by bespoke packages of video and audio resource libraries. Virtual clinics will offer one to one, remote consultations through a triaging service. The members will have access to the vast range of educational material on specific topics.

Additionally, remote in-person consultations will also be available not only to the professionals but also to service users throughout the course of spinal injury management, from the acute stage to lifelong follow-up care. The application will also target those who are rehabilitated at home or in a non-specialist rehabilitation centre.

**Figure 3.**
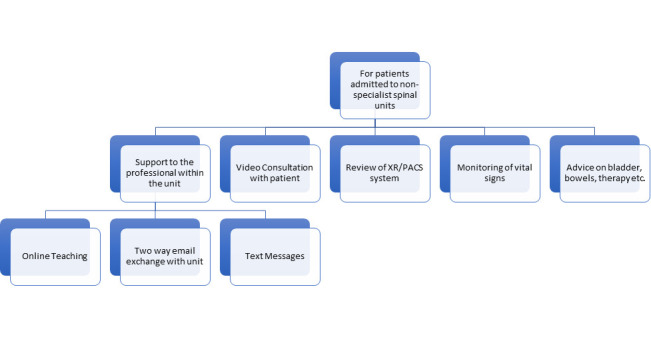
Use of technology in Acute Rehabilitation non-specialist spinal units.

### ESTABLISH PATHWAYS FOR THE USE OF DIGITAL HEALTH TECHNOLOGIES IN REHABILITATION

#### EMERGENCY AND ACUTE CARE

Although this takes place in the Trauma Centre or MTN, there is no reason why a rehabilitation team cannot be involved in the early decision-making process either in-person or remotely using digital health technology. Technology would allow interaction from the rehabilitation team at a more senior level, if required. Thus, the decision to stabilise a spine could be taken by a team of doctors (surgeons and physicians) looking not only at mending the broken or crumbling bones but dealing with the consequences of the spinal shock and dysfunction of the sympathetic and parasympathetic nervous systems that results from neurological insult. Early care could be taken of the bladder, bowels, skin, and other systems. Coordination for the rehabilitation input should be provided by a consultant in spinal injuries and rehabilitation medicine who liaises with their team of specialist nurses, physiotherapists, occupational therapists, speech and language therapists, psychologists, counsellors and other professionals for comprehensive management. This can also include ‘conservative' management (i.e., without surgery) to deal with certain types of spinal injuries.

**Figure 4.**
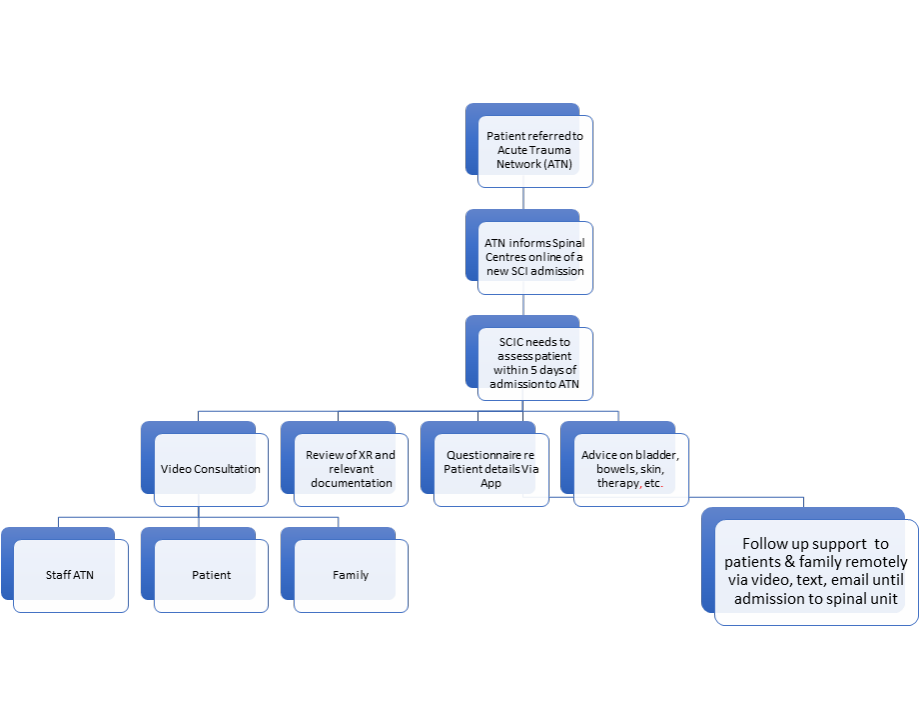
Use of technology for acute spinal cord injury (SCI) management.

#### ACUTE AND SUBACUTE CARE PATHWAYS

A team of highly skilled and experienced multidisciplinary professionals is poised to develop a comprehensive digital training/education programme on SCI and its management. The service will be of benefit for the professionals in Major Trauma Network, General Practice Surgeries and other non-specialist medical or rehabilitation units to assist them in early recognition of the condition, prompting early referral to the SCICs. Also, training and education would enable the professionals from these organisations to care for the individuals with SCI appropriately while they await a bed at the SCIC.

The envisioned web portal will be a primary resource on evidence-based practice, information on guidelines, care pathways and protocols of SCI management. At any stage during the acute management of an individual, the specialists in rehabilitation can also arrange ‘one to one' remote consultation with the acute care specialists via the application, allowing timely access to specialist input and better clinical outcomes.

Our project will focus not only on the professionals but also individuals with SCI and their family members. For those with recent injuries and their family members experiencing much anxiety while waiting for a bed at SCIC, this application will offer information, advice and support in the early stages.

**Figure 5:**
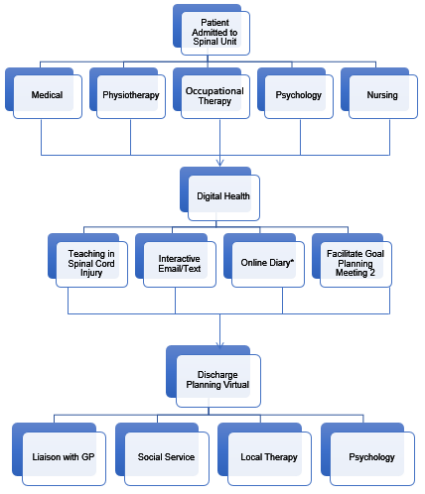
Use of technology in acute rehabilitation.

Note: (*) To allow family to visit at appropriate times; (2) Allows local clinical/social teams to attend.

#### REHABILITATION AND LONG-TERM CARE

This service can offer continuous professional development training modules for the staff at SCIC and tailor-made teaching programmes to the professionals in rehabilitation centres not specialised in the management but caring for individuals with SCI who may be admitted due to lack of specialist beds. The teaching modules will comprise of a series of videos and virtual talks along with live chats and consultations. It will have the potential to provide quicker access to SCI specialists, saving time and cost of travel. The web portal will offer teachings to individuals on aspects of spinal cord injury care.

#### LONG-TERM FOLLOW-UP

The service will also target individuals post-discharge from SCIC, offering follow-up care via the application. Virtual clinics and remote consultations with an expert via the web portal can support SCIC outreach teams, enhancing their effectiveness in the community.

**Figure 6.**
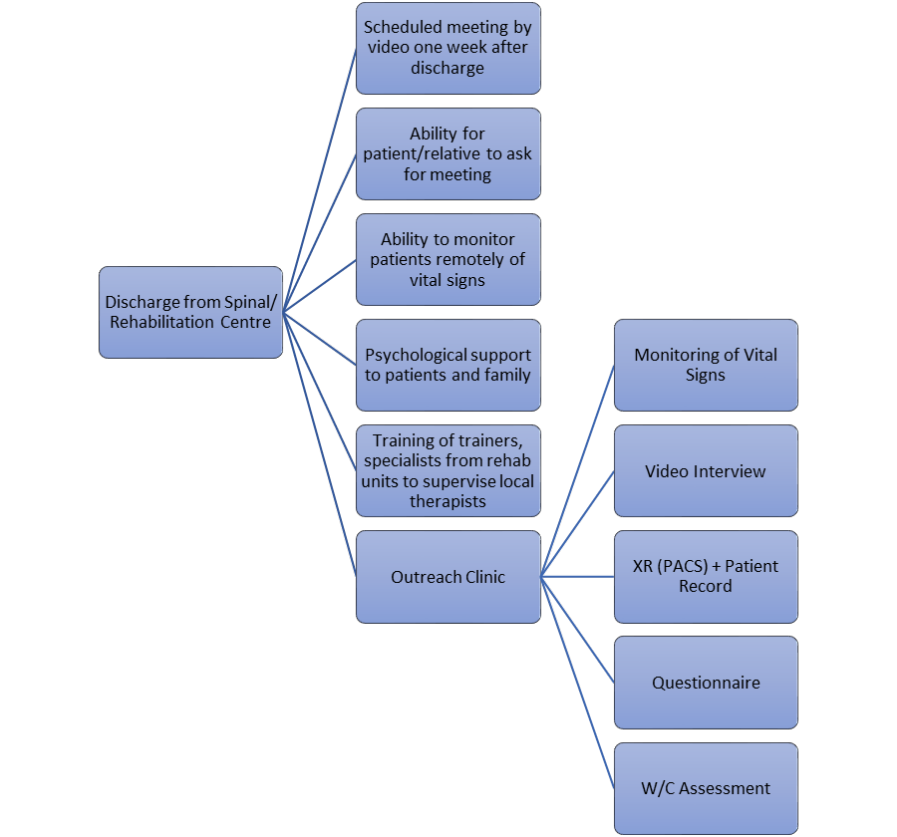
Use of technology at discharge into the community.

The application has the potential to provide lifetime follow-up care in the community - including ongoing medical and therapy assessments and advice, remote monitoring of vital signs, and early recognition of secondary medical complications like pressure injuries. Individuals with SCI can have consultations with specialists via virtual clinics through a triage service on the application. Those who require psychological support could request input from a specialist counsellor or clinical psychologist.

In the longer term, the application will work for the professionals and individuals with SCI in an interlinked manner. It will enable spinal outreach teams at SCIC to work in partnership with local district nurses and community care teams (who do not otherwise have the expertise in SCI management) in monitoring early signs of complications specific to SCI.

Virtual clinics can be an option in the future as the yearly plain X rays and renal ultrasound can be made available via PACS links or secure cloud to the spinal unit outreach teams. Community teams can complement any missing data. As a result, Spinal Injury Centres can always monitor their service users in the community effectively and without the need for travel, saving a significant cost on transport. Technology would allow the input from various specialists simultaneously and during another episode of care if necessary, without the need for travel.

The service aims to provide permanent, regular, and effective follow-up care for the spinal cord injury population in the UK, offering peer support, ongoing advice and education by the experts in the field. We make the assumption that the care provided will be cost-effective and help reduce the number of secondary complications post-discharge, thereby reducing readmissions and reliance on the SCIC.

#### DATA COLLECTION AND RESEARCH

People with SCI are scattered across the wide catchment areas. Our experience is that they may not always report to their spinal teams the local admissions for bony fractures, chest infections and other medical conditions. Digital Health Rehabilitation can be a useful tool to capture such data with an in-built questionnaire and help with future research opportunities.

#### CATCHMENT AREA

Digital Health Rehabilitation can reach out to geographically remote areas that are otherwise difficult to access by the specialist professionals, allowing benefits of expertise and skills to a larger population without geographical bias. The technology will be developed to make the device accessible in remote areas with poor internet facilities and lower grade smartphones. Rehabilitation services can expand the continuity of therapeutic input by providing skilled and personalised therapy interventions at the convenience of the individual's home (Peretti et al., 2017). It has the capacity to consistently provide input at all stages of rehabilitation, throughout an individual's lifetime, fulfilling the ideology of ‘rehabilitation from injury to grave' in modern times.

#### EXPANDING TO OUTSIDE OF THE UK

There is potential for market opportunities and expanding the service outside of the UK as many European countries have also cut funding to health care, social care, and non-governmental organisations in the last 5-10 years, causing growing inequality between rural and urban areas in accessing health services and community-based rehabilitation (“ICT for Health and i2010,” 2006). The World Health Organisation's report on SCI admitted that worldwide people with SCI have substantial unmet needs for follow-up services and primary care, post-rehabilitation period (Bickenbach et al., 2013). The unmet primary care needs include health promotion, prevention services and medical treatment

We initially considered a universal solution that would apply to the whole world; however, a recent meeting by World Federation of Neurorehabilitation on Telerehabilitation (WFNR, 2019) in Crotone Italy made us reconsider our initial plans. It had become clear that legislation in many countries was being revised as digital health was perceived as a threat to private practice and earnings. However, more recently with the emergence of COVID 19 the acceptance of digital health appears to be increasing.

#### SCOPE OF TECHNOLOGY

Increasingly sophisticated technologies are being deployed in some countries to provide home-based exercise, monitoring, diet and medication compliance tracking, robotics-based treatment, and other more dynamic interventions with the use of artificial intelligence. Our vision is to apply these sophisticated technologies to rehabilitation medicine through Digital Health Rehabilitation and create a robust and sustainable model/pathway of spinal cord injuries management.

#### PROVIDING INDIVIDUALISED CARE

Since Digital Health Rehabilitation relies on the complex interaction between professionals, technology, and processes, its success also lies in the successful functioning of these three factors. It is often mentioned that ‘technology can only get as clever as the user' and hence Digital Health Rehabilitation only aims to support and empower the professionals and not in any way replace them in the future. Provision of individualised care is the aim. What we have listed above is by no means a comprehensive list.

## CONCLUSION

For many decades, the UK spinal centres managed to provide lifelong care to the SCI population, but today, they are under stress and, therefore, once discharged, a large number of individuals are almost forgotten in the community. Sir Ludwig Gutman's vision for spinal cord injury ‘rehabilitation from injury to grave' that founded the very principle of SCI management is challenged in the modern world, wherein health systems are increasingly not able to cope with the demand. Digital Health Rehabilitation can be a solution to many problems faced by the health care system in the UK and the rest of the world, at all stages of the journey of an individual with SCI from injury to return into the Community. Our project also aims to create good quality evidence on the use of Digital Health Rehabilitation in spinal cord injury management in the UK and establish processes and protocols in its implementation. The purpose of this Commentary is to encourage adoption of the concept by those who commission health services and to convince private investors to support development of the project.
